# Challenges in distinguishing functional proteins from polyproteins in databases: implications for drug discovery

**DOI:** 10.1093/bib/bbae012

**Published:** 2024-02-01

**Authors:** Ariadna Llop-Peiró, Gerard Pujadas, Santiago Garcia-Vallvé

**Affiliations:** Departament de Bioquímica i Biotecnologia, Universitat Rovira i Virgili, Research Group in Cheminformatics & Nutrition, Tarragona, Spain; Departament de Bioquímica i Biotecnologia, Universitat Rovira i Virgili, Research Group in Cheminformatics & Nutrition, Tarragona, Spain; Departament de Bioquímica i Biotecnologia, Universitat Rovira i Virgili, Research Group in Cheminformatics & Nutrition, Tarragona, Spain

**Keywords:** molecular databases, polyproteins, SARS-CoV-2, drug research

## Abstract

This opinion article addresses a major issue in molecular biology and drug discovery by highlighting the complications that arise from combining polyproteins and their functional products within the same database entry. This problem, exemplified by the discovery of novel inhibitors for the severe acute respiratory syndrome coronavirus 2 (SARS-CoV-2) main protease, has an influence on our ability to retrieve precise data and hinders the development of targeted therapies. It also emphasizes the need for improved database practices and underscores their significance in advancing scientific research. Furthermore, it emphasizes the need of learning from the SARS-CoV-2 pandemic in order to improve global preparedness for future health crises.

Databases such as UniProt [1] and ChEMBL [2] are essential tools in drug research that provide resources for finding information about proteins and identifying molecules that can bind to them and affect their activity. However, a significant challenge arises when these databases have to deal with polyproteins that undergo proteolytic cleavage to produce distinct functional proteins. This issue is particularly prominent in viruses, such as human immunodeficiency virus (HIV) and severe acute respiratory syndrome coronavirus 2 (SARS-CoV-2), where a single polyprotein precursor produces multiple functional proteins. UniProt, following the ‘one gene, one protein’ hypothesis [3], groups all products of a single gene under a single UniProtKB/Swiss-Prot entry. While conceptually sound, this practice makes it challenging for databases like ChEMBL to collect specific data for these proteins. This is especially evident in the context of viral polyproteins, given their prevalence in viral genomic structures.

The development of antiviral drugs against SARS-CoV-2 provides a notable example of this challenge [4]. In SARS-CoV-2 and other coronaviruses, the ORF1ab gene encodes the polyproteins pp1a and pp1ab [5]. Pp1ab includes pp1a, and its synthesis requires a ribosomal frameshift. Both polyproteins are subsequently processed by the main protease (M-pro) and papain-like protease (PLpro) to yield 15 non-structural proteins, which include M-pro, PLpro, an RNA-dependent RNA polymerase and other proteins involved in the transcription and replication of the viral genome. The Uniprot entry P0DTD1 contains the sequence of the replicase polyprotein pp1ab from SARS-CoV-2, and the proteins derived from the hydrolysis of pp1a and ppa1b, such as M-pro, are found in the PTM/Processing section of this entry. In the ChEMBL database, which is a manually curated database of bioactive molecules, the entry CHEMBL4523582 contains bioactivity data for molecules targeting any of the replicase polyprotein pp1ab products, including M-pro. This organization makes it difficult to precisely collect specific information, for example the IC_50_ values, about bioactive molecules that target the M-pro from SARS-CoV-2.

Combining functional components of polyproteins within a single database entry makes accurate data retrieval and analysis difficult. This approach lacks the detail needed for precise molecular research, making it difficult to separate data specific to different proteins. The difficulties outlined herein are not unique to SARS-CoV-2, but are representative of a broader issue encountered with polyproteins across various biological contexts. To solve this issue, UniProt now employs a feature identifier, which includes the PRO prefix, separated by an underscore from a 6 to 10-digit number, to provide a unique and stable identifier for each of the proteins derived from a polyprotein [4, 6, 7]. As a result, the SARS-CoV-2 M-pro has the unique identifier P0DTD1-PRO_0000449623. Some specialized protein-related databases and tools, including IntAct, SWISS-MODEL, Reactome and PDBe-KB, use these feature identifiers defined by UniProt. In contrast, other databases, such as KEGG, use their own identifiers to address this problem. However, other databases like ChEMBL, DrugBank, PDB, PubChem and BindingDB have not yet implemented an effective solution to separate the information of distinct proteins derived from a polyprotein, making it difficult to collect data specific to these proteins and potentially leading to manual curation errors. Solving this problem is critical for advancing drug discovery, particularly in antiviral drug development. Lessons learned from the COVID-19 pandemic must guide future efforts to provide a more effective response to future health threats.

**Key Points**

- Annotating polyproteins poses a challenge within molecular databases.
- The development of SARS-CoV-2 M-pro inhibitors as an example.
- The use of unique identifiers for functional proteins of polyproteins is a potential solution.

## Data Availability

Details about the public data we used have been incorporated in the article. No additional data have been generated.

## Notes

**Ariadna Llop-Peiró** is currently undertaking her Ph.D. in the Cheminformatics & Nutrition Research Group at Rovira i Virgili University (URV), where her primary focus is the search for new antiviral drugs to address potential future pandemics. Prior to this, she obtained a Bachelor's Degree in Biotechnology from URV and a Master's Degree in Cytogenetics and Reproductive Biology from the Autonomous University of Barcelona (UAB). During her time as a student trainee in the Neurobiotechnology group at the Institute for Bioengineering of Catalonia (IBEC), Ariadna contributed to the analysis of the Blood-Brain Barrier. Her work contributed to explore therapeutic transport mechanisms, with the ultimate goal of establishing effective ways to administer drugs for the treatment of infections.

**Gerard Pujadas** graduated in Chemistry from the Universitat de Barcelona in 1992 and completed his PhD in Chemistry at the Universitat Rovira i Virgili (URV, Tarragona) in 1998. During his early career as a researcher, he became acquainted with the subjects of bioinformatics and protein structure analysis, recognizing their importance in sequence evolution. In 2001 he made a post-doctoral stay at Prof. Richard Haser's lab at the Institut de Biologie et Chimie des Protéines (Lyon, France). Following this post-doctoral experience, he returned to the URV, securing a permanent position at the Biochemistry and Biotechnology Department in September 2002. Since then, his main contributions to science have been the development of: (1) several virtual screening workflows for predicting molecules that act as anti-inflammatories (i.e., IKK-2 inhibitors) or anti-diabetics (i.e., DPP-IV inhibitors and PPAR-gamma partial agonists); (2) tools for validating virtual screening workflows (i.e., DecoyFinder); and (3) tools for validating the coordinates of drug-target complexes (i.e., VHELIBS). Since April 2014, he has led the research group “Cheminformatics & Nutrition” at the URV.

**Santiago Garcia-Vallvé** obtained his Bachelor's degree in Chemistry (1995) and completed his PhD in Biochemistry in 1999 at the Universitat Rovira i Virgili (URV) in Tarragona, Spain. Since 2003 he has a permanent position in the Department of Biochemistry and Biotechnology at URV. Over the period from 1995 to 2009, his research primarily focused on bioinformatics, sequence analysis, and molecular evolution. Notable contributions included the development of databases, servers, and tools for predicting horizontally transferred genes and highly expressed genes in prokaryotic complete genomes. In 2001, he was a visitor at the European Bioinformatics Institute (EBI) in Cambridge, UK. Presently, he is a member of the Cheminformatics & Nutrition Research Group at URV. His ongoing research endeavors are concentrated on leveraging cheminformatics tools to identify new bioactive compounds for specific targets. Simultaneously, he is dedicated to pioneering new tools that enhance virtual screening performance. In summer 2016, he was a visitor at the Department of Chemistry, Cambridge University, UK. Since 2020, a significant portion of the research conducted by the Cheminformatics & Nutrition Research Group has been directed towards the study of SARS-CoV-2, analyzing its mutations, and identifying inhibitors of the main protease (M-pro).
